# Supplementary material for: Visual perceptual learning generalizes to untrained effectors
Source: J Vis. 2021 Mar 8;21(3):10. doi: 10.1167/jov.21.3.10 (PMC7961118; doi:10.1167/jov.21.3.10)
Supplement: Supplement 1 [file jovi-21-3-10_s001.pdf]

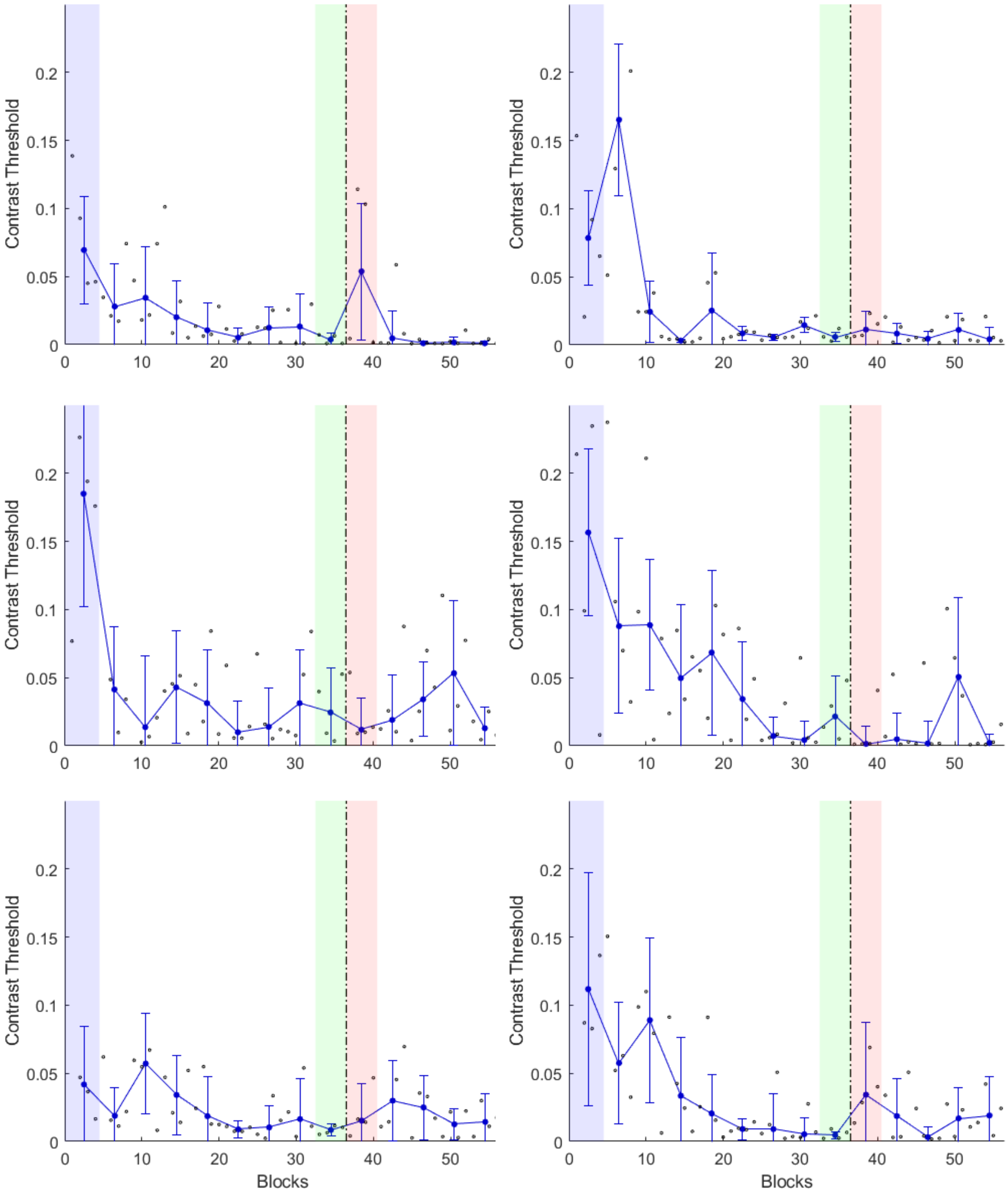

Figure S1 Learning curves for each observer (n = 6) in Experiment 1. The dashed line represents the change in experimental phase. In phase one (left of the dashed line), the observer reported the direction of the motion with a saccade. In phase two (right of the dashed line), the observer reported the direction of the motion with a manual response (keyboard). Small black open circles represent the contrast threshold for each block (125 trials). Blue dots represent the contrast threshold for each training session/day (median threshold for 4 blocks). Error bars show the standard deviation from the mean contrast threshold for each training session/day. Shaded regions represent the time periods for each threshold measurement. Baseline threshold (blue) was computed as the threshold during the first day of training with the saccade. The training threshold was computed as the threshold during the last day of training with the saccade (green). The transfer threshold was computed as threshold during the first day of training with the manual response (pink).

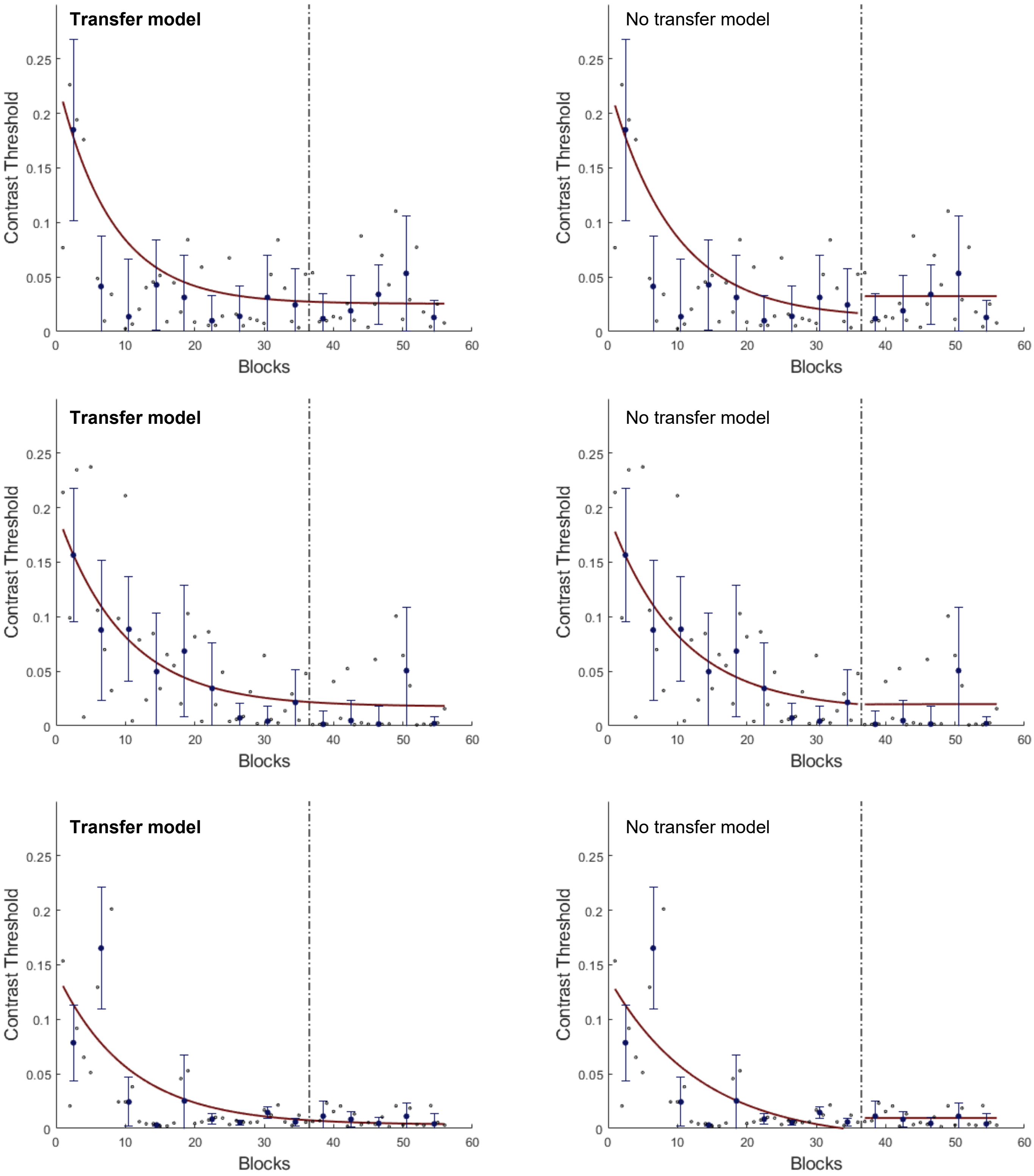

Transfer model
No transfer model
Transfer model
No transfer model
Transfer model
No transfer model
Contrast Threshold
Blocks
0
0.05
0.1
0.15
0.2
0.25
0
10
20
30
40
50
60

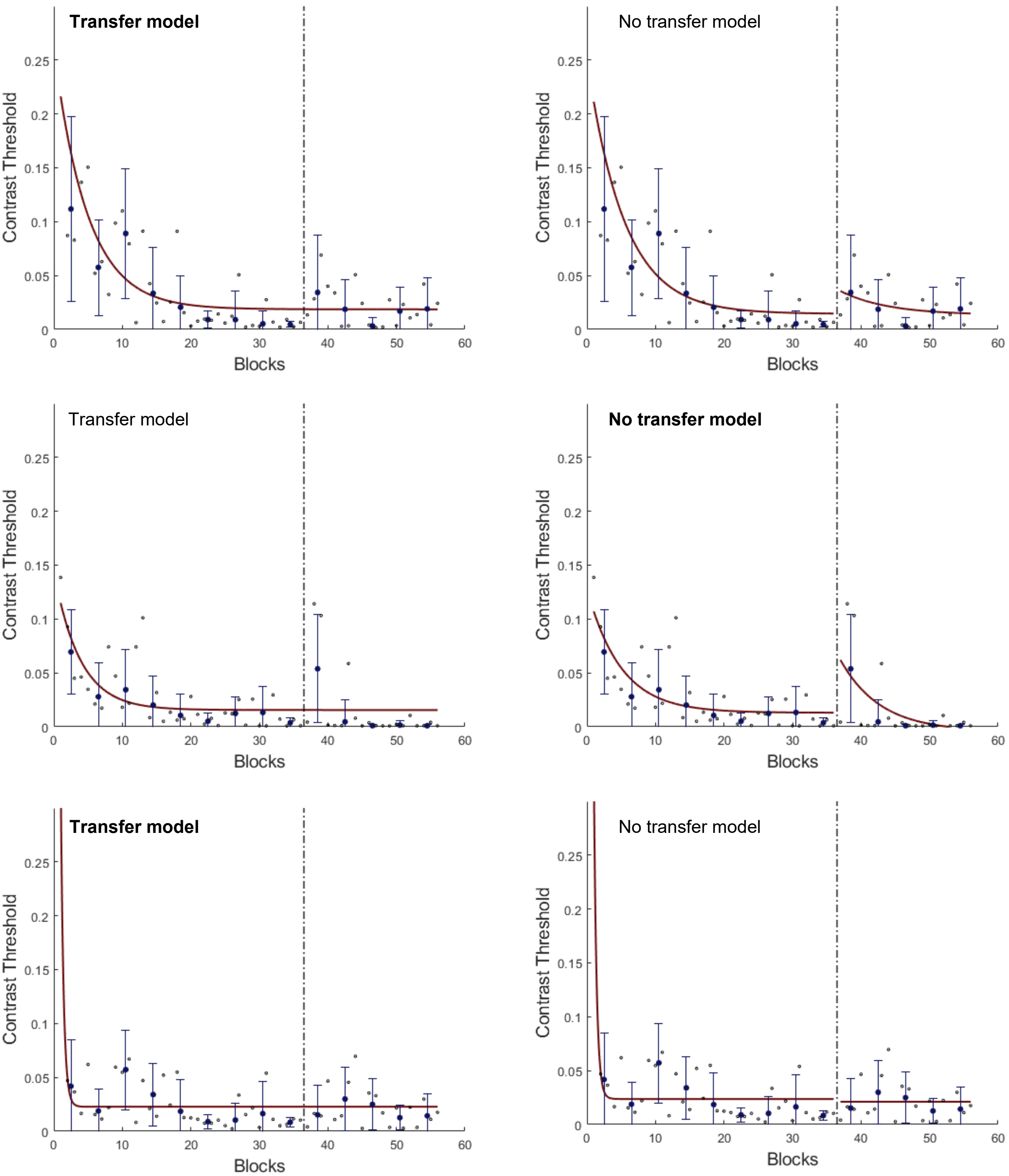

Figure S2 Data and model fitting for each observer in Experiment 1. Each row represents the two models for one observer. The dashed vertical line represents a change in the experimental phase (from saccade to manual response). Small black open circles represent the contrast threshold for each block (125 trials). Blue dots represent the contrast threshold for each training session (median threshold for 4 blocks). Error bars show the standard deviation from the mean contrast threshold for each training session. The red curve represents the model fitting curve in the transfer model (left) and the no transfer model (right). In 5

of the observers, the transfer model significantly fit the data better than the no transfer model (ΔBIC = 7.5, range [7,8]). The better model is shown in bold for each observer.

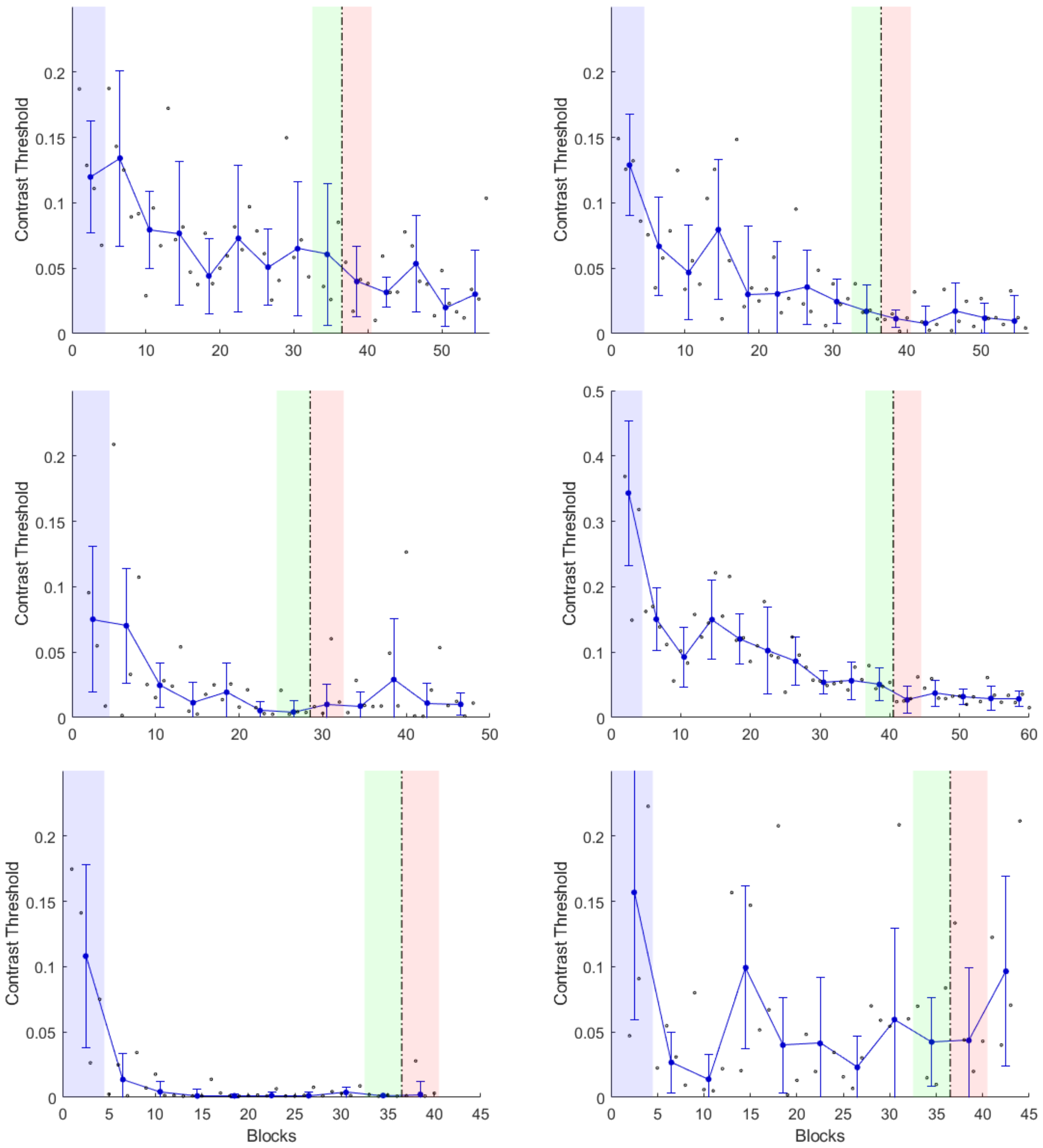

Figure S3 Learning curves for each observer (n = 6) in Experiment 2. The dashed line represents the change in experimental phase. In phase one (left of the dashed line), the observer reported the direction of the motion with a manual response (keyboard). In phase two (right of the dashed line), the observer reported the direction of the motion with a saccade. Small black open circles represent the contrast threshold for each block (125 trials). Blue dots represent the contrast threshold for each training session/day (median threshold for 4 blocks). Error bars show the standard deviation from the mean contrast threshold for each training session/day. Shaded regions represent the time periods for each threshold measurement. Baseline threshold (blue) was computed as the threshold during the first day of training with the manual response. The training threshold was computed as the threshold during the last five days of training with the manual response (green). The transfer threshold was computed as threshold during the five days of training with the saccade (pink). Due to the COVID-19 pandemic, two observers (bottom row) were unable to complete the five days of training in phase 2 in Experiment 2.

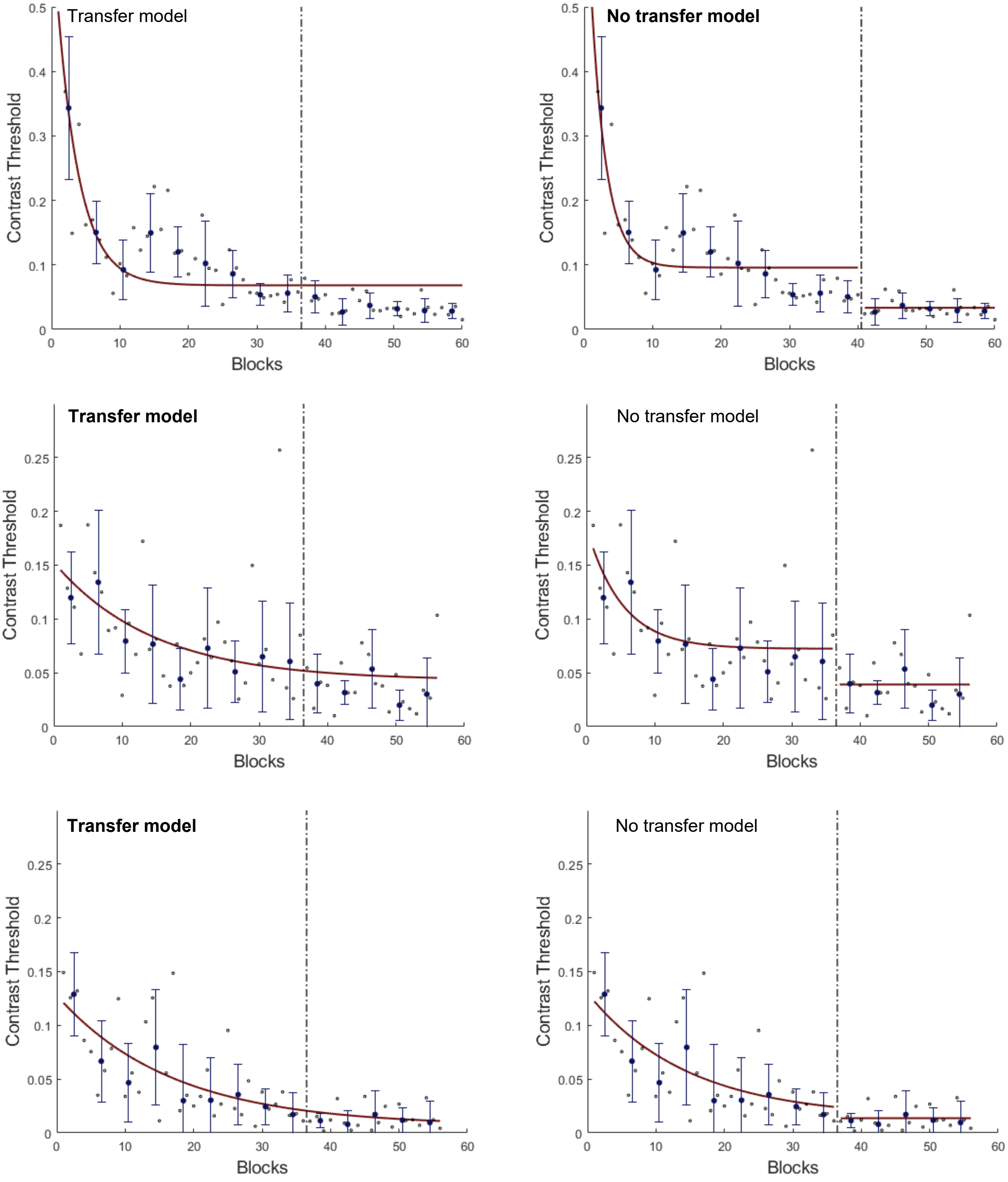

Transfer model
No transfer model
Transfer model
No transfer model
Transfer model
No transfer model
Contrast Threshold
Blocks

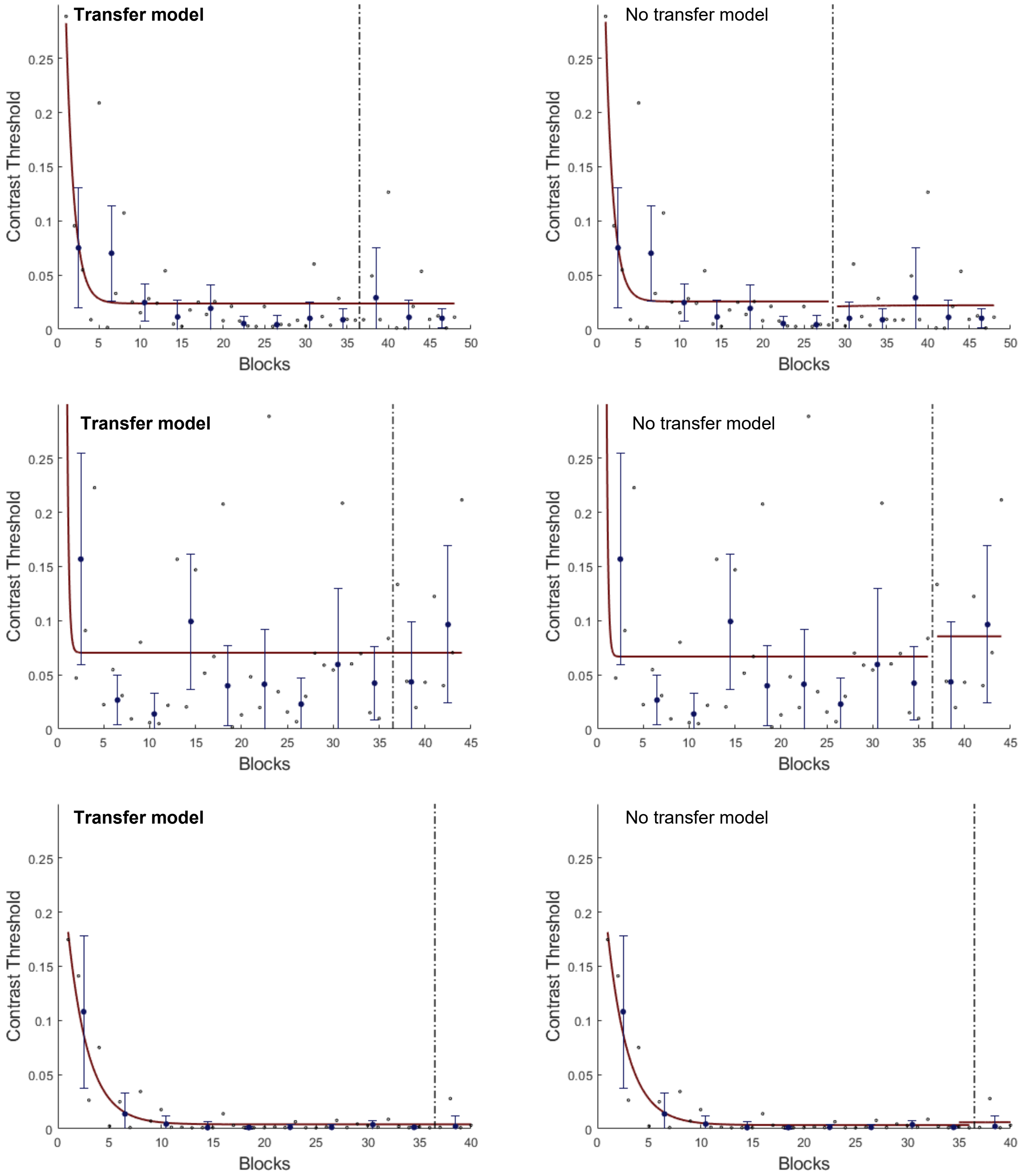

Figure S4 Data and model fitting for each observer in Experiment 2. Each row represents the two models for one observer. The dashed vertical line represents a change in the experimental phase (from manual response to saccade). Small black open circles represent the contrast threshold for each block (125 trials). Blue dots represent the contrast threshold for each training session (median threshold for 4 blocks). Error bars show the standard deviation from the mean contrast threshold for each training session. The red curve represents the model fitting curve in the transfer model (left) and the no transfer model (right). In 5 of the observers, the transfer model significantly fit the data better than the no transfer model (ΔBIC = 7,

range [4,8]). The better model is shown in bold for each observer. For the one observer whose data was better fitted by the no transfer model (top row), the performance in the second phase of the experiment improved, indicative of transfer. Due to the COVID-19 pandemic, two observers (bottom row) were unable to complete the five days of training in phase 2 in Experiment 2.
